# Melanoma LAMP-2C Modulates Tumor Growth and Autophagy

**DOI:** 10.3389/fcell.2018.00101

**Published:** 2018-08-29

**Authors:** Liliana Pérez, Anthony L. Sinn, George E. Sandusky, Karen E. Pollok, Janice S. Blum

**Affiliations:** ^1^Virus Persistence and Dynamics Section, Vaccine Research Center, National Institute of Allergy and Infectious Diseases, National Institutes of Health, Bethesda, MD, United States; ^2^In Vivo Therapeutics Core, Indiana University Melvin and Bren Simon Cancer Center, Indiana University School of Medicine, Indianapolis, IN, United States; ^3^Department of Pathology, Indiana University School of Medicine, Indianapolis, IN, United States; ^4^Department of Pharmacology and Toxicology, Indiana University School of Medicine, Indianapolis, IN, United States; ^5^Department of Pediatrics, Indiana University School of Medicine, Indianapolis, IN, United States; ^6^Department of Microbiology and Immunology, Indiana University School of Medicine, Indianapolis, IN, United States

**Keywords:** LAMP-2, LAMP-2C, macroautophagy, chaperone-mediated autophagy, melanoma, tumor

## Abstract

Autophagy plays critical but diverse roles in cellular quality control and homeostasis potentially checking tumor development by removing mutated or damaged macromolecules, while conversely fostering tumor survival by supplying essential nutrients during cancer progression. This report documents a novel inhibitory role for a lysosome-associated membrane protein, LAMP-2C in modulating autophagy and melanoma cell growth *in vitro* and *in vivo*. Solid tumors such as melanomas encounter a variety of stresses *in vivo* including inflammatory cytokines produced by infiltrating lymphocytes directed at limiting tumor growth and spread. Here, we report that in response to the anti-tumor, pro-inflammatory cytokine interferon-gamma, melanoma cell expression of *LAMP2C* mRNA significantly increased. These results prompted an investigation of whether increased melanoma cell expression of LAMP-2C might represent a mechanism to control or limit human melanoma growth and survival. In this study, enhanced expression of human LAMP-2C in melanoma cells perturbed macroautophagy and chaperone-mediated autophagy in several human melanoma lines. *In vitro* analysis showed increasing LAMP-2C expression in a melanoma cell line, triggered reduced cellular LAMP-2A and LAMP-2B protein expression. Melanoma cells with enhanced LAMP-2C expression displayed increased cell cycle arrest, increased expression of the cell cycle regulators Chk1 and p21, and greater apoptosis and necrosis in several cell lines tested. The increased abundance of Chk1 protein in melanoma cells with increased LAMP-2C expression was not due to higher *CHEK1* mRNA levels, but rather an increase in Chk1 protein abundance including Chk1 molecules phosphorylated at Ser345. Human melanoma cell xenografts with increased LAMP-2C expression, displayed reduced growth in immune compromised murine hosts. Melanomas with high LAMP-2C expression showed increased necrosis and reduced cell density upon histological analysis. These results reveal a novel role for LAMP-2C in negatively regulating melanoma growth and survival.

## Introduction

Basal levels of autophagy are critical to cellular homeostasis by eliminating malfunctioning organelles and long-lived proteins (Levine and Kroemer, 2008). Autophagy increases with nutrient deprivation and hypoxia (Levine and Kroemer, 2008). Defects in autophagy impact several diseases, including cancer (Levine and Kroemer, 2008; Morselli et al., 2009; Choi, 2012). However, the role of autophagy in cancer development is complex. While basal autophagy may function as a tumor suppressor, increased or induced autophagy may contribute to tumor survival during cancer progression (Morselli et al., 2009; Choi, 2012).

Two forms of autophagy, MA and CMA are detectible in human cells and upregulated in many tumors (Morselli et al., 2009; Kon et al., 2011; Choi, 2012). MA increases with cell nutrient stress and temporally wanes as CMA increases and is sustained. During nutrient or growth factor deprivation, MA and CMA are upregulated to promote cell survival by recycling building blocks, modulating bioenergetics, and shifting metabolism. During MA, cytoplasmic macromolecules and organelles are sequestered inside autophagosomes, which fuse with lysosomes to promote content degradation. Basal levels of MA may prevent tumor development by modulating chromosome stability and removing mutated proteins and damaged mitochondria (Morselli et al., 2009; Choi, 2012). However, with tumor progression and exposure to metabolic stresses, MA is induced to recycle nutrients, favor tumor survival and resistance to anti-cancer therapies (Morselli et al., 2009; Choi, 2012). During CMA HSC70 and HSP90, capture cytoplasmic proteins for selective translocation into lysosomes for degradation (Agarraberes and Dice, 2001). CMA is upregulated in many tumors including melanoma, breast, and lung cancers (Kon et al., 2011; Saha, 2012; Zhou et al., 2016). CMA relies on a LAMP-2A to translocate cytoplasmic proteins into lysosomes (Cuervo and Dice, 1996). Hyper-expression of LAMP-2A is observed in tumors, while disrupting LAMP-2A expression slows tumors growth and metastasis (Kon et al., 2011; Zhou et al., 2016).

LAMP-2 is a highly glycosylated protein localized in acidic lysosomal and endosomal compartments. Alternative splicing generates three isoforms LAMP-2A, LAMP-2B, and LAMP-2C, which differ primarily in the sequence of their transmembrane and cytosolic tail (Eskelinen et al., 2005). LAMP-2A and LAMP-2B are constitutively expressed by all cells, while LAMP-2C has a much more limited tissue distribution (Perez et al., 2016). LAMP-2A, the receptor for CMA may modulate aging and tumor growth (Cuervo and Dice, 1996, 2000; Kon et al., 2011; Perez et al., 2016). LAMP-2B is involved in lysosome biogenesis and MA (Nishino et al., 2000). Mutations in LAMP-2B have been reported to disrupt autophagosome maturation. LAMP-2C can facilitate DNA and RNA translocation into lysosomes, while enhanced LAMP-2C expression inhibits CMA in human B lymphoblasts (Fujiwara et al., 2013a,b; Perez et al., 2016). Yet, little is known about LAMP-2C function in tumor cell growth and autophagy.

*In vivo*, tumors such as melanomas encounter infiltrating immune cells producing pro-inflammatory cytokines, which can induce stress and limit tumor growth. While melanoma cells express relatively low levels of LAMP-2C compared to LAMP-2A and LAMP-2B, as shown here exposure of these cells to the cytokine IFN-γ significantly increased *LAMP2C* mRNA abundance. By contrast, only marginal changes in *LAMP2A* mRNA expression and no difference in *LAMP2B* mRNA abundance were detected in IFN-γ treated melanoma cells. These cytokine-induced changes suggested that LAMP-2C could potentially play a role in regulating tumor cell survival and responses to stress. In this study, we explored the role of LAMP-2C in the growth and survival of human melanoma cells using a rodent xenograft model. Human melanoma cells were transfected to increase LAMP-2C protein expression. In the melanoma cell line DM331, ectopic expression of LAMP-2C resulted in decreased expression of LAMP-2A and LAMP-2B proteins. CMA was diminished in cells with increased LAMP-2C, as indicated by the increased abundance of several proteins typically targeted for degradation by CMA including Chk1, IκBα, and p21 (Cuervo et al., 1998; Park et al., 2015; Zhang et al., 2018). Significant reductions in MA were also detected in melanomas with increased LAMP-2C expression based on analysis of MA flux and autophagosome abundance. Ectopic expression of LAMP-2C altered melanoma cell growth *in vitro* and cell cycle progression with increased apoptosis and necrosis detectable in several melanoma cell lines. These changes in the cell cycle may be related to the greater abundance of Chk1 and phospho-Chk1 as well as p21 in melanomas with increased LAMP-2C. *In vivo*, human melanoma cells with increased LAMP-2C displayed reduced growth and increased necrosis compared with the parental melanoma cell line. This study demonstrates a novel role for LAMP-2C in melanoma growth and offers innovative strategies for targeting subcutaneous melanoma.

## Materials and Methods

### Cell Lines and Transfection

The human melanoma cell line DM331 provided by Dr. V. Engelhard (University of Virginia) was maintained in RPMI-1640 with 5% FBS, 50 U/ml penicillin, 50 μg/ml streptomycin, and 1% L-glutamine (Slingluff et al., 2000). The human melanoma cell line SLM2-Mel provided by Dr. W. J. Storkus (University of Pittsburgh School of Medicine) was maintained in the same media with 0.1% β-mercaptoethanol (Haque et al., 2002). Melanoma cell lines were transfected using Xfect Transfection Reagent (Clontech, Mountain View, CA, United States). Control vectors or vectors encoding human *LAMP2C* have been described (Perez et al., 2016).

### Reverse Transcription Polymerase Chain Reaction (RT-PCR)

To detect *LAMP2* or *GAPDH* transcript expression, cellular RNA was extracted using RNeasy Mini Kit (Qiagen, Valencia, CA, United States) and cDNA was generated using the High-Capacity cDNA Reverse Transcription Kit (Applied Biosystems, Foster City, CA, United States). Primers for *LAMP2* and *GAPDH* amplification were described (Perez et al., 2016). *LAMP2* cDNA was amplified using 2X ReddyMix PCR Master Mix (Thermo Fisher Scientific, Waltham, MA, United States) for 35 cycles. *GAPDH* cDNA was amplified for 30 cycles. PCR products were resolved by agarose gel.

### Real-Time Quantitative PCR (qPCR)

qPCR was performed using custom Taqman primers for *LAMP2A, LAMP2B*, and *LAMP2C* (Perez et al., 2016) or commercial primers *CDKN1A, CHEK1, CTSA, CTSB, CTSD, NFKBIA, TP53, ACTB, GAPDH* or *18S*, and the 7500 Fast RT-PCR System from Applied Biosystems. Gene expression was normalized to *ACTB, GAPDH* or *18S* mRNA levels and presented as a relative fold change compared with control samples or presented as mRNA expression relative to *18S* mRNA levels. For analysis of fold changes in mRNA, if differences of less than twofold were detected, trends in expression were noted rather than statistical significance.

### Western Blotting

Cells were lysed on ice for 30 min with RIPA buffer, protease inhibitor cocktail ± phosphatase inhibitor cocktail. Cell lysate proteins (80 μg) were resolved on SDS-PAGE and transferred to nitrocellulose for western blots. Blots were quantitated by densitometry using ImageJ (NIH, Bethesda, MD, United States) and normalized to cellular actin. Antibodies against LAMP-2A (Cat #ab18528), LAMP-2B (Cat #ab18529), HSP90 (Cat #ab13494), and cathepsin A (Cat #ab79590) were from Abcam (Cambridge, MA, United States). Chk1 (Cat #2360), phospho-Chk1 (Ser345) (Cat #2341), IκBα (Cat #4814), phospho-IκBα (Ser32/36) (Cat #9246), LC3B (Cat #2775), and histone H3 (Cat #3638) were from Cell Signaling Technology (Danvers, MA, United States). LAMP-2 (Cat #H4B4-c) was from DSHB (Iowa City, IA, United States) and HSC70 (Cat #ADI-SPA-815) from Enzo Life Sciences (Farmingdale, NY, United States). Anti-Myc Tag (Cat #05-724) and cathepsin D (Cat # IM03) were from EMD Millipore (Billerica, MA, United States). Cathepsin B (Cat # sc-13985), p53 (Cat # sc-126), and p21 (Cat # sc-756) were from Santa Cruz Biotechnology (Santa Cruz, CA, United States). Actin (Cat # MS-1295-P0) was from Thermo Fisher Scientific.

### Interferon-Gamma Treatment

DM331 cells were incubated 24 h at 37°C with 400 or 2000 units (IU) of recombinant human IFN-γ (R&D Systems, Minneapolis, MN, United States). Cells were harvested and *LAMP2* mRNA was measured by qPCR.

### MA Analysis

To detect MA flux, cells were incubated for 16 h at 37°C ± 20 μM CQ (Sigma-Aldrich, St. Louis, MO, United States) (Mizushima and Yoshimori, 2007; Mizushima et al., 2010; Klionsky et al., 2012). Western blotting was used to detect cellular LC3I and LC3II. Cellular LC3I and LC3II protein levels were normalized relative to actin protein levels to account for protein sample loading. MA flux was determined by subtracting the relative ratio of LC3II/actin in untreated cells from the relative ratio of LC3II/actin for CQ treated cells. To monitor MA in real time within live cells, melanoma cells were incubated 4 h at 37°C with media ± serum. Vesicles produced during MA in normal or starvation conditions were stained using CYTO-ID Autophagy detection kit (Enzo Life Sciences) and analyzed by flow cytometry (Guo et al., 2015).

### Lysosomal Proteases or Calpain Inhibition

To detect changes in LAMP-2A protein levels, cells were incubated 18 h at 37°C ± 20 μM CQ or 10 μM calpeptin (EMD Millipore). Samples were resolved on SDS-PAGE and analyzed by western blotting.

### Apoptosis Assay

For detection of apoptotic and necrotic cells, real time analysis of caspase-3 and caspase-7 activity was detected using CellEvent Caspase-3/7 Green Flow Cytometry Assay Kit (Invitrogen, Carlsbad, CA, United States). During apoptosis, caspase-3 and caspase-7 are activated and able to cleave a cell permeable fluorogenic substrate DEVD peptide. The bright fluorogenic signal produced by caspase-3 and caspase-7 activity indicates apoptotic cells. Cells positive for AAD dead cell stain help separate live from dead cells. Samples were analyzed by flow cytometry.

### Subcellular Fractionation

Cytoplasmic and nuclear proteins were extracted using NE-PER Nuclear and Cytoplasmic Extraction Reagents (Thermo Fisher Scientific) following manufacturer’s recommended instructions. Samples were resolved on SDS-PAGE and analyzed by western blotting.

### Cell Cycle Analysis

Cells were fixed with 70% cold ethanol (-20°C) for 1 h at 4°C, washed with ice-cold PBS, incubated 15 min at 37°C with 100 μg/ml RNase A (Sigma-Aldrich), and then stained 30 min at room temperature with 50 μg/ml of propidium iodide (Sigma-Aldrich). Samples were analyzed by flow cytometry.

### [^3^H] Thymidine Incorporation

Cells were incubated with [^3^H] thymidine for 8 h at 37°C. Thymidine incorporation was quantified using Wallac 1450 Microbeta Plus liquid scintillation counter (Perkin Elmer, Shelton, CT, United States).

### Reactive Oxygen Species Analysis

Basal ROS were measured by incubating cells with 5 μM CellROX Deep Red Reagent (Thermo Fisher Scientific, Waltham, MA, United States) for 30 min at 37°C. This cell-permeant dye is non-fluorescent while in a reduced state, and fluoresces upon oxidation by ROS. Samples were analyzed by flow cytometry.

### Proteasome Assay

Proteasome activity was determined using Proteasome-Glo Chymotrypsin-Like Cell-Based Assay (Promega, Madison, WI, United States) (Moravec et al., 2009). Cells were trypsinized and plated according to the manufacturer’s recommended instructions. Cells were incubated with Proteaseome-Glo Cell Based Reagent to deliver the substrate (Succinyl-LLVY-aminoluciferin) into the cytoplasm of the cells. Aminoluciferin, which is the substrate for luciferase, is released following cleavage of this peptide substrate by the proteasome. Luciferase consumption of aminoluciferin results in a luminescent signal that is proportional to the amount of proteasome activity (Moravec et al., 2009). Luminescence was detected using SpectraMax M5 Microplate Reader (Molecular Devices, Sunnyvale, CA, United States). Studies have indicated the specificity of this assay in multiple cultured cell lines in detecting changes in proteasome activity (Moravec et al., 2009).

### Xenograft Studies

Female NOD.Cg-*Prkdc^*scid*^Il2rg^*tm1Wjl*^*/SzJ mice 6–8 weeks of age were obtained from the *In Vivo* Therapeutics Core of the Indiana University and injected in the flanks with 5 × 10^5^ melanoma cells. Animals were monitored two to three times a week following tumor implantation to detect changes in health and weight. Tumor size was measured biweekly as length (mm) × width (mm) × width (mm)/2 to obtain a measure of volume in mm cubed. All animals were terminated 20 days after tumor implantation, and tissues and palpable tumors were collected for analysis.

### Ethics Statement

Mice were maintained in specific pathogen-free conditions under conditions approved by the Institutional Animal Care and Use Committee of Indiana University School of Medicine and the Guide for the Care and Use of Laboratory Animals.

### Tissue Processing and Staining

Tissues were fixed in 10% neutral-buffered formalin at 4°C for 24 h followed by processing and paraffin embedding. Five-micrometer sections were cut and stained for hematoxylin and eosin (HE) or phospho-Histone H3 (EMD Millipore, Cat #06-570).

### Assessment of Necrosis and Phospho-Histone H3 (pH3) Positivity

The Aperio ScanScope CS system whole slide digital imaging system (Leica Biosystems, Buffalo Grove, IL, United States) was used for imaging slides at 20X. Necrosis was determined by quantifying cells with nuclear fragmentation in randomly selected fields of primary tumors. As a measure of mitosis, five hot spots were selected per slide and cells positive for pH3 staining were quantified using the Positive Pixel Count V9 algorithm of Aperio ImageScope software (Leica Biosystems, Buffalo Grove, IL, United States). pH3 positivity represents pH3 positively stained cells divided by the total number of cells in the selected areas.

### Statistical Analysis

Data were analyzed by two-way ANOVA or by two-tailed, unpaired Student’s *t*-test using GraphPad Prism 6.0 (GraphPad Software, San Diego, CA, United States). A value of *p* < 0.05 or less was considered significant for all experiments. Error bars indicate SD unless noted otherwise.

## Results

### Expression of LAMP-2C in Human Melanoma Cells

**FIGURE 1:**
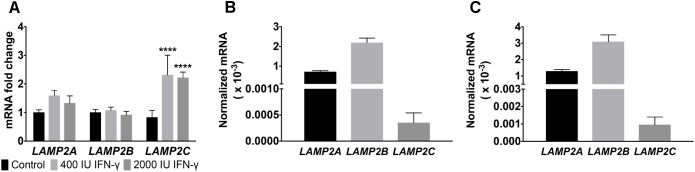
*LAMP2* expression in melanomas during IFN-γ treatment. **(A)** DM331 cells treated for 24 h with 400 or 2000 IU of IFN-γ. Gene expression of *LAMP2A, LAMP2B*, and *LAMP2C* were analyzed by qPCR. mRNA levels were normalized to *ACTB* expression with the expression of each isoform set equal to one for control cells without cytokine exposure. **(B)** Endogenous mRNA levels of *LAMP2* isoforms in DM331 cells were quantitated relative to *18S* mRNA levels. **(C)** Endogenous mRNA levels of *LAMP2* isoforms in SLM2-Mel cells. Gene expression was quantitated relative to *18S* mRNA levels. Data were analyzed by two-way ANOVA. ^∗∗∗∗^*p* < 0.0001 (*n* = 3).

Therapeutic treatment of many cancers including melanoma with IFN-γ, is well documented (Zaidi and Merlino, 2011). IFN-γ negatively impacts tumor growth and alters the expression of multiple genes (Zaidi and Merlino, 2011). Exposure of tumors to IFN-γ can induce cell stress marked in some cases by increased cellular ROS production, upregulation of the DNA damage response, as well as enhanced cell senescence and death (Hubackova et al., 2016). Prior work had shown that exposure to toll-like receptor ligands, immune mediators often associated with infection, alters *LAMP2* isoforms mRNA expression in human B lymphoblasts (Perez et al., 2016). To address whether differential regulation of *LAMP2* isoforms is observed in human melanomas, we exposed melanoma cells to IFN-γ. A twofold to threefold induction of *LAMP2C* mRNA was observed upon melanoma cells exposure to IFN-γ with very modest changes in the more abundant *LAMP2A* and no induction of *LAMP2B* (**Figure 1A**). These results suggest that *LAMP2C* expression can be upregulated by cytokine stress in human melanomas.

**FIGURE 2:**
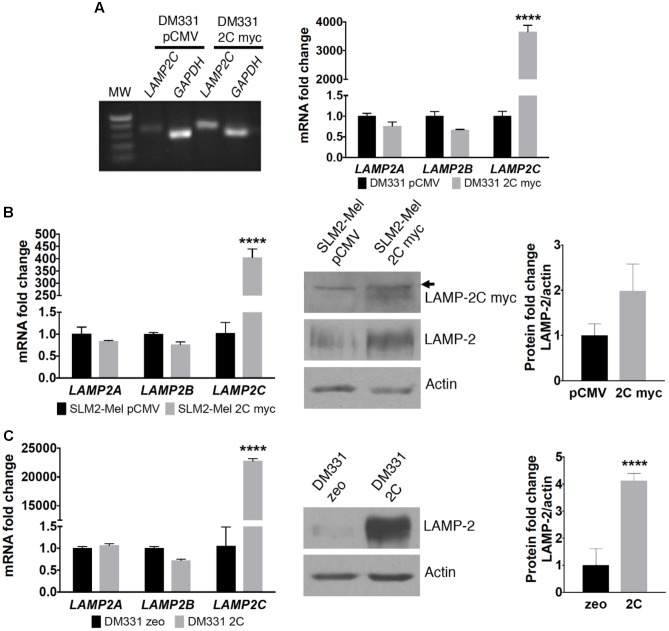
LAMP-2 expression in human melanoma cell lines transfected with LAMP-2C. **(A)** DM331 cells were transfected with an empty vector (pCMV) or a plasmid encoding for C-terminal myc tagged *LAMP2C*. RT-PCR analysis for *LAMP2C* overexpression was detected in an agarose gel. mRNA levels of *LAMP2A, LAMP2B*, and *LAMP2C* transcripts were analyzed by qPCR and normalized to *18S* expression. To detect relative changes in the expression of each isoform, the normalized expression of each isoform was set equal to one in DM331 pCMV cells. **(B)** SLM2-Mel cells were transfected with an empty vector (pCMV) or a plasmid encoding for C-terminal myc tagged *LAMP2C*. mRNA levels of *LAMP2A, LAMP2B*, and *LAMP2C* in these cells were analyzed by qPCR and normalized to *ACTB* expression. The relative expression of each isoform was set equal to one for SLM2-Mel pCMV control cells. Cell lysates were probed for the c-myc tagged LAMP2C or total LAMP2 protein with actin used as a control for sample loading. Arrow indicates non-specific protein band detected with anti-myc antibody. **(C)** DM331 cells were transfected with an empty vector (zeo) or a plasmid encoding for *LAMP2C* with no tag sequence. mRNA levels of *LAMP2A, LAMP2B*, and *LAMP2C* in these cells were analyzed by qPCR and normalized to *GAPDH* expression. To examine relative changes in each isoform, the expression of individual isoforms was set to one for the DM331 zeo control cells. Cell lysates were probed for total LAMP2 protein with actin used as a control for sample loading. Data were analyzed by two-way ANOVA or by two-tailed, unpaired Student’s *t*-test. ^∗∗∗∗^*p* < 0.0001 (*n* = 2–3).

The hierarchy of endogenous *LAMP2* mRNA expression (*LAMP2B* > *LAMP2A* > *LAMP2C*) was consistent among two distinct human melanoma cell lines, DM331 and SLM2-Mel (**Figures 1B,C**). Given the low basal levels of *LAMP2C* mRNA in each melanoma cell line, this isoform was ectopically expressed in each cell line to examine its impact on autophagy, cell growth and survival (**Figure 2**). LAMP-2 isoforms can be detected using commercial antibodies that recognize conserved epitopes, but individual isoform analysis can be challenging given their structural homology. To circumvent the absence of antibodies against LAMP-2C, melanoma cells (DM331 or SLM2-Mel) were transfected with a plasmid encoding C-terminal myc tagged *LAMP2C* yielding DM331 2C myc or SLM2-Mel 2C myc cells (**Figures 2A,B**). As a control, the parental cell lines were transfected with an empty vector to produce DM331 pCMV or SLM2-Mel pCMV cells (**Figures 2A,B**). As an additional control, DM331 cells were also transfected with a distinct empty vector (DM331 zeo) or a plasmid encoding untagged *LAMP2C* (DM331 2C) to ensure the myc tag was not impacting function (**Figure 2C**). Increased *LAMP2C* mRNA was detected in melanoma cells transfected with the *LAMP2C* plasmid (**Figure 2**). Higher levels of ectopic *LAMP2C* mRNA were detected in DM331 cells compared to the SLM2-Mel cells, regardless of myc tag addition (**Figure 2**). While there was no significant change in *LAMP2A* mRNA levels with ectopic LAMP-2C expression in cells, a slight reduction was observed in mRNA levels of *LAMP2B* (**Figure 2**).

**FIGURE 3:**
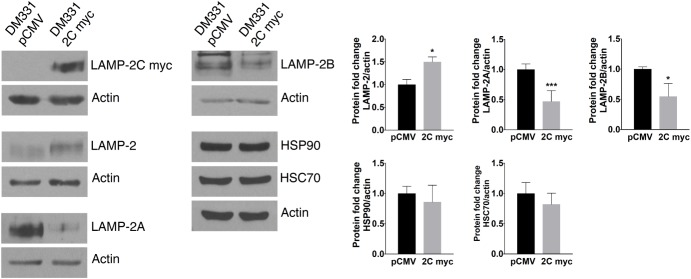
LAMP-2C ectopic expression in melanoma cells altered LAMP-2A and LAMP-2B protein expression. LAMP-2C myc, total LAMP-2, LAMP-2A, LAMP-2B, HSC70, HSP90, and actin were detected in DM331 pCMV and DM331 2C myc cells. The normalized expression of each protein was set equal to one for DM331 pCMV cells for relative comparison with protein levels in DM331 2C myc cells. Data were analyzed by two-tailed, unpaired Student’s *t*-test. ^∗^*p* < 0.05 and ^∗∗∗^*p* < 0.001 (*n* = 3).

Western blot analysis of melanoma cells revealed similar electrophoretic migration of ectopic LAMP-2C and other LAMP-2 isoforms on SDS-PAGE (**Figures 2B,C, 3**). LAMP-2 isoforms are translated as polypeptides of approximately 42 kDa, with glycosylation of these isoforms yielding proteins which migrate as diffuse bands on SDS-PAGE with an apparent molecular mass of 120 kDa. The diffuse appearance and similar electrophoretic migration of LAMP-2C ectopically expressed with or without a myc tag in melanomas, was consistent with a high degree of glycosylation observed with other LAMP-2 isoforms. Cellular levels of total LAMP-2, detected with an antibody recognizing all isoforms, were increased 1.5- to 4-fold in melanoma cells likely due to the increase in *LAMP2C* mRNA (**Figures 2B,C, 3**). Notably, the expression of both LAMP-2A and LAMP-2B proteins was reduced about 50% in cells with increased LAMP-2C (**Figure 3**). Cellular levels of CMA chaperones HSC70 and HSP90 were unperturbed by increased LAMP-2C (**Figure 3**). These findings suggest that increased LAMP-2C expression in melanoma cells may affect cellular levels of LAMP-2A and LAMP-2B proteins.

**FIGURE 4:**
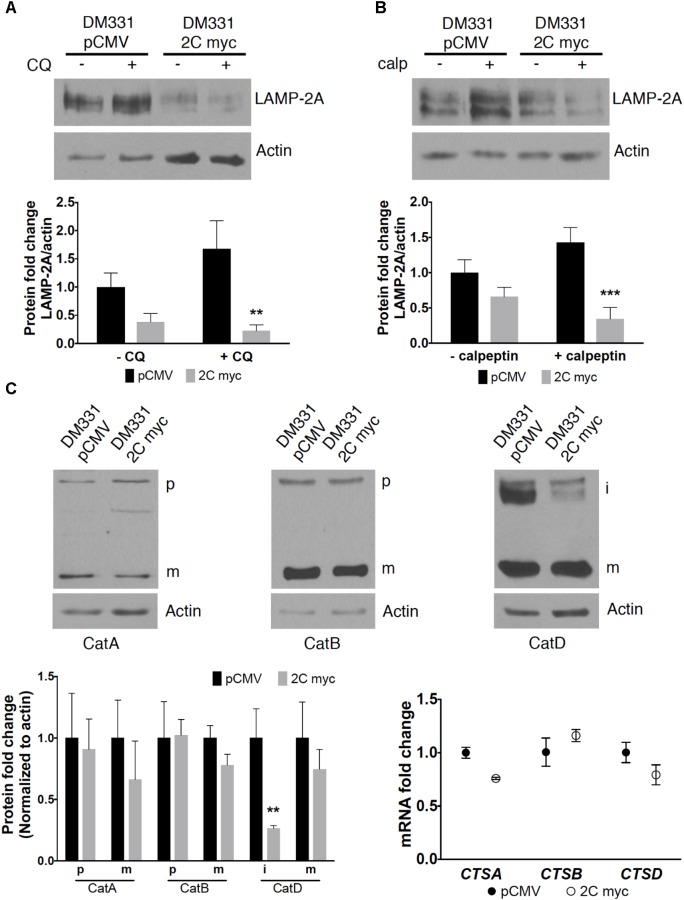
Changes in LAMP-2A protein expression were not due to increased cathepsin or calpain abundance in melanomas expressing LAMP-2C. DM331 pCMV and DM331 2C myc cells were incubated overnight at 37°C with ±20 μM CQ **(A)** or 10 μM calpeptin (calp) **(B)** to inhibit lysosome proteases or calpain activity, respectively. LAMP-2A levels were detected by western blotting, evaluated by densitometry, and normalized to actin protein levels. LAMP-2A levels were calculated relative to DM331 pCMV cells cultured without CQ or calpeptin. **(C)** Maturation and gene expression of lysosome proteases cathepsin A (CTSA), cathepsin B (CTSB), and cathepsin D (CTSD) was evaluated in cells overexpressing LAMP-2C. Lysates were resolved by SDS-PAGE and probed to detect the precursor (p), intermediate (i), or mature (m) form of cathepsin A, cathepsin B, and cathepsin D. Protein expression was quantified by densitometry and levels were normalized to actin levels. mRNA levels of *CTSA, CTSB*, and *CTSD* transcripts were analyzed by qPCR and normalized to *18S* expression. Measurements in **(A–C)** are relative values calculated by setting the results obtained for DM331 pCMV cells equal to one for comparison to DM331 2C myc cells. Data were analyzed by two-way ANOVA. ^∗∗^*p* < 0.01, and ^∗∗∗^*p* < 0.001 (*n* = 3).

Tumors have been manipulated using molecular approaches to reduce constitutive *LAMP2A* mRNA expression to impact cell growth (Kon et al., 2011; Saha, 2012; Zhou et al., 2016). Here, the reduction in LAMP-2A protein abundance with ectopic LAMP-2C expression suggested post-translational regulation of this isoform’s expression. A lysosomal serine protease cathepsin A and a cytoplasmic cysteine protease calpain I regulate LAMP-2A protein stability and turnover (Cuervo et al., 2003; Villalpando Rodriguez and Torriglia, 2013). To examine whether melanoma cell LAMP-2C expression impacts proteolytic turnover of LAMP-2A, DM331 2C myc cells were incubated with CQ, a weak base which prevents cathepsin A activation in acidic organelles, or with calpeptin, a cell permeable calpain inhibitor. The addition of these agents to control DM331 pCMV cells, with low endogenous LAMP-2C, slightly increased steady state LAMP-2A protein abundance (**Figures 4A,B**). Yet in melanoma cells with high LAMP-2C expression, treatment with these inhibitors unexpectedly promoted an even greater reduction in cellular LAMP-2A protein levels. CQ treatment neutralizes lysosome, endosome, and autophagosome pH, reducing the activity of multiple enzymes including proteases functional at low pH. We examined several lysosomal cathepsins to determine if LAMP-2C expression increased the abundance and maturation of these enzymes to active proteases, possibly explaining the observed decrease in melanoma cell levels of LAMP-2A protein with ectopic LAMP-2C expression. Cellular levels of mature and precursor forms of lysosomal proteases cathepsin A and cathepsin B were unchanged in melanoma cells by ectopic LAMP-2C. Expression of the mature cathepsin D (30 kDa) protein was also not statistically different with ectopic LAMP-2C expression in cells, while cathepsin D immature precursors (46 kDa and 52 kDa forms) were significantly decreased in cells with high LAMP-2C expression (**Figure 4C**). The 30 kDa and 46 kDa forms of cathepsin D are functional aspartyl proteases. Quantitative analysis of transcripts for cathepsin genes *CTSA, CTSB*, and *CTSD* corroborated that ectopic expression of LAMP-2C in melanoma cells did not increase the expression of these lysosomal enzyme mRNAs (**Figure 4C**). Rather a slight decrease in *CTSA* and *CTSD* mRNA was detected in cells with ectopic LAMP-2C. Thus, the decreased abundance of LAMP-2A observed in melanoma cells with high LAMP-2C expression, was not linked to an increased cellular accumulation of these three cathepsin proteases. Together, these results suggest increased LAMP-2C expression in melanoma cells perturbs steady state levels of LAMP-2A and LAMP-2B, each of which has been implicated in regulating autophagy pathways.

### LAMP-2C Expression Impacts CMA and MA

**FIGURE 5:**
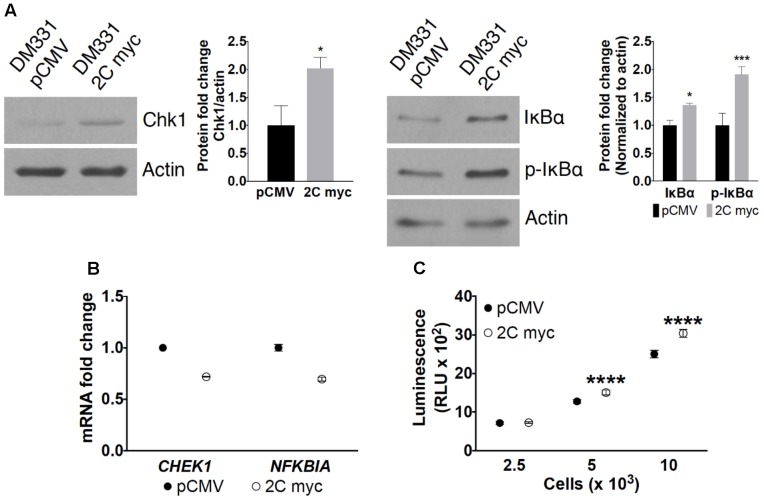
Effect of LAMP-2C expression on CMA substrates. **(A)** Cellular levels of CMA substrates Chk1, IκBα, and p-IκBα in DM331 pCMV and DM331 2C myc cells were examined by western blotting. Relative protein levels were calculated by setting the normalized expression to one for DM331 pCMV cells. **(B)** mRNA levels of *CHEK1* and *NFKBIA* transcripts were analyzed by qPCR and normalized to *ACTB* expression. mRNA levels in DM331 pCMV cells were normalized and set to one. **(C)** Proteasome activity was measured using the Proteasome-Glo Chymotrypsin-Like Cell-Based Assay. Cells were incubated with a substrate succinyl-LLVY-aminoluciferin which penetrates into the cytoplasm. This substrate is cleaved by the proteasome to release aminoluciferin which is released from cells. Luciferase is added to these cells, cleaving aminoluciferin to a luminescent product detectable using a luminometer. Data were analyzed by two-way ANOVA or by two-tailed, unpaired Student’s *t*-test. ^∗^*p* < 0.05, ^∗∗∗^*p* < 0.001, and ^∗∗∗∗^*p* < 0.0001 (*n* = 3).

Impaired CMA can alter intracellular accumulation of select cytoplasmic proteins targeted for degradation by this pathway. Steady state levels of two well-described CMA protein substrates, the cell cycle regulator Chk1 and the inhibitor of NF-κB signaling pathway IκBα, were examined in melanoma cells with ectopic LAMP-2C expression (Cuervo et al., 1998; Park et al., 2015). Elevated levels of Chk1 and total or phosphorylated IκBα were observed in DM331 2C myc melanoma cells, suggesting disruptions in the proteolytic turnover of these proteins via CMA (**Figure 5A**). Increased cellular expression of Chk1 and IκBα was not due to higher *CHEK1* and *NFKBIA* mRNA transcripts, again consistent with CMA disruption in melanoma cells with high LAMP-2C expression (**Figure 5B**). Changes in autophagy can impact cytoplasmic protein degradation by the proteasome (Park and Cuervo, 2013). CMA substrates Chk1 and IκBα can be diverted to the proteasome in some cell types (Alkalay et al., 1995; Zhang et al., 2005). We quantitated proteasome proteolytic activity in DM331 2C myc cells using a specific proteasome substrate, succinyl-LLVY-aminoluciferin, delivered selectively into the cytoplasm of melanoma cells. Proteasome activity was not reduced in cells with ectopic LAMP-2C expression but rather slightly increased compared to control cells (**Figure 5C**). These data suggest that LAMP-2C myc expression in melanoma cells disrupts CMA and increased cellular protein levels of several CMA substrates.

**FIGURE 6:**
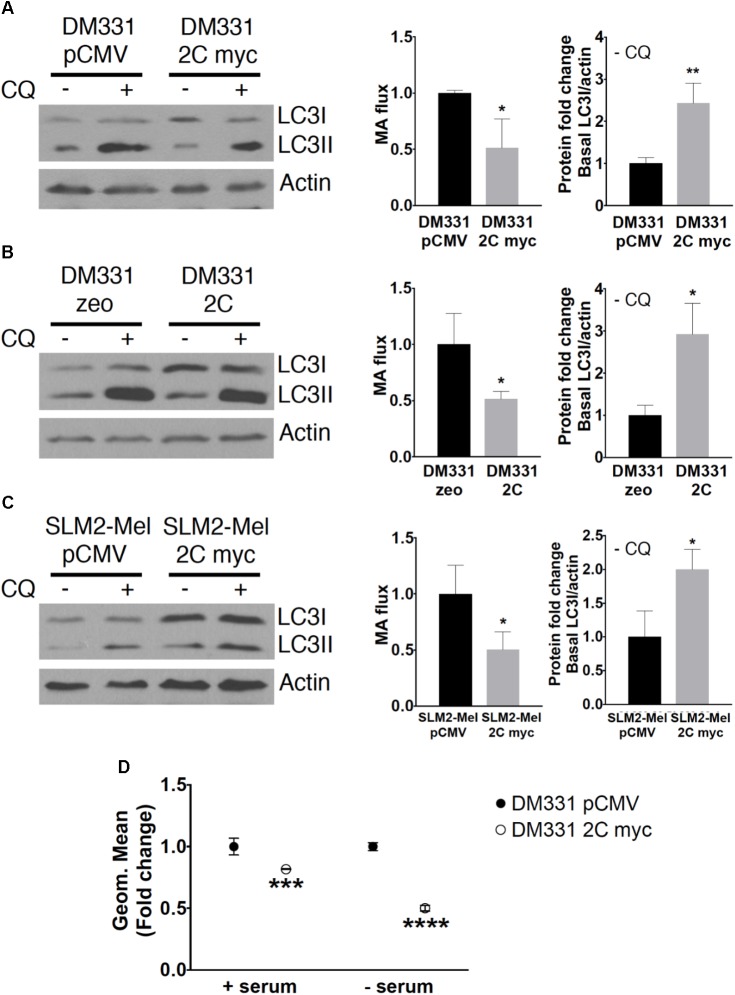
MA was diminished in melanomas with increased LAMP-2C. **(A–C)** DM331 or SLM2-Mel cells were incubated overnight with ±20 μM CQ, an inhibitor of lysosome acidification, to monitor autophagosome formation and turnover. To measure MA flux, the conversion of LC3I to LC3II was detected and normalized to cellular actin levels. MA flux was quantified to detect changes in cellular LC3II levels ± CQ using the equation MA flux = LC3II/actin in CQ treated cells – LC3II/actin in control cells. The relative levels of LC3I/actin in each cell line grown without CQ provides a measure of basal LC3I protein accumulation prior to its enzymatic conversion to LC3II during MA. **(D)** DM331 pCMV and DM331 2C myc cells were incubated with media ± serum, stained with CYTO-ID, and MA monitored by flow cytometry. The geometric mean was set equal to one for DM331 pCMV cells for relative comparison to the geometric mean in DM331 2C myc cells. Data were analyzed by two-way ANOVA or by two-tailed, unpaired Student’s *t*-test. ^∗^*p* < 0.05, ^∗∗^*p* < 0.01, ^∗∗∗^*p* < 0.001, and ^∗∗∗∗^*p* < 0.0001 (*n* = 3).

LAMP-2B is required for efficient cellular MA, thus changes in MA were examined in melanoma cells with increased LAMP-2C (Nishino et al., 2000). The intracellular abundance and stability of LC3 I and II are used to monitor MA (Mizushima and Yoshimori, 2007; Mizushima et al., 2010; Klionsky et al., 2012). During MA, cytoplasmic LC3I is lipidated, and converted to LC3II, which associates with autophagosomes. LC3II is then proteolyzed upon autophagosome maturation marking a full cycle of MA. An accurate measure of this autophagy pathway can be obtained through analysis of MA progression or flux (Klionsky et al., 2012). Changes in LC3I and LC3II protein levels were detected in each of the melanoma cells with ectopic LAMP-2C in the presence or absence of CQ, the latter which neutralizes autophagosome acidification to slow LC3II degradation during MA. Monitoring the relative LC3II (LC3II/actin) levels in cells treated with CQ and subtracting the relative LC3II (LC3II/actin) abundance in cells without CQ, offers a measure of MA progression or flux (Klionsky et al., 2012). MA flux was diminished in DM331 cells with increased LAMP-2C myc (**Figure 6A**). Decreased MA flux was observed in DM331 cells expressing untagged LAMP-2C and a distinct cell line, SLM2-Mel 2C myc (**Figures 6B,C**). Consistent with the flux analysis suggesting disruptions in MA in melanoma cells with ectopic LAMP-2C, the relative levels of LC3I (basal LC3I/actin) were increased in untreated melanoma cells. While relative LC3I abundance in cells does not measure MA, the detected accumulation of LC3I may suggest a slowing or disruption in early stages of MA in the context of reduced flux. As an alternate approach to evaluate cellular MA in these melanoma cells, DM331 2C myc were treated with a dye CYTO-ID that fluoresces upon delivery into autophagosomes. Cellular stresses, such as nutrient starvation, promote an increase in CYTO-ID accumulation in newly forming autophagosomes (Guo et al., 2015). DM331 pCMV and DM331 2C myc cells were incubated ± serum and autophagosomes stained using CYTO-ID to evaluate MA. MA was reduced in melanoma cells with LAMP-2C cultured in serum as detected by flow cytometry (**Figure 6D**). Reductions in MA were apparent in serum nutrient starved DM331 cells with increased LAMP-2C expression compared to control cells (**Figure 6D**). Tumor cells may encounter a variety of stresses *in vivo* including limitations in nutrient availability, oxygen deficiency, and inflammatory mediators. Experiments here suggest that ectopic LAMP-2C expression in melanoma cells reduces MA under basal and stress conditions. Thus, increased LAMP-2C expression in melanoma cells results in disruptions in cellular MA.

### Ectopic LAMP-2C Expression Perturbs Cell Cycle and Survival

**FIGURE 7:**
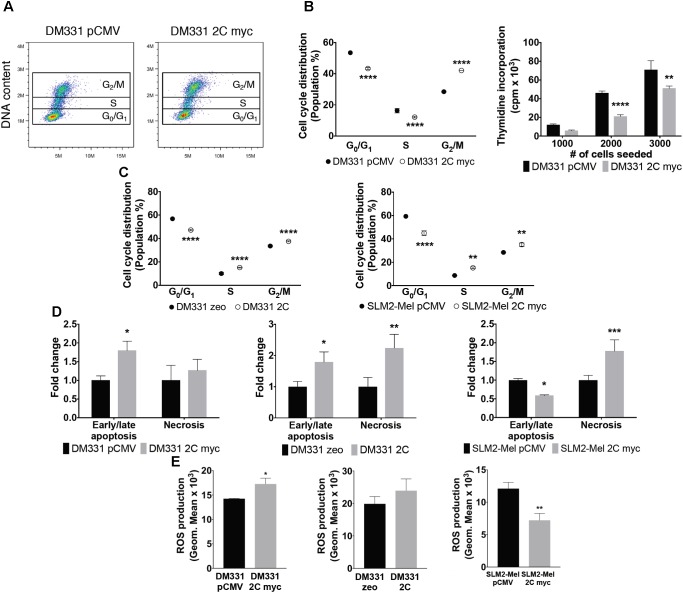
Reduced survival and altered cell cycle in melanomas expressing LAMP-2C. **(A)** Apoptosis and necrosis were examined by incubating DM331 pCMV and DM331 2C myc cells with a fluorogenic substrate specific for activated caspase-3 and caspase-7 in apoptotic cells and AAD dead cell stain to detect necrotic cells. Apoptotic and necrotic cells were detected by flow cytometry. **(B)** Representative dot plot graph of the cell cycle distribution of DM331 pCMV and DM331 2C myc cells. Cell cycle distribution was evaluated by staining DNA content of DM331 pCMV and DM331 2C myc cells with propidium iodide and quantified by flow cytometry. **(C)** Cell cycle distribution of DM331 zeo and DM331 2C was evaluated by staining DNA content with propidium iodide and quantified by flow cytometry. **(D)** Cell cycle distribution was analyzed by staining the DNA content of SLM2-Mel pCMV and SLM2-Mel 2C myc with propidium iodide and detected by flow cytometry. **(E)** To examine basal ROS production, melanomas with and without ectopic LAMP-2C expression were incubated for 30 min at 37°C with 5 μM CellROX Deep Red Reagent and monitored by flow cytometry. Data were analyzed by two-way ANOVA or by two-tailed, unpaired Student’s *t*-test. ^∗^*p* < 0.05, ^∗∗^*p* < 0.01, ^∗∗∗^*p* < 0.001, and ^∗∗∗∗^*p* < 0.0001 (*n* = 2–3).

Autophagy pathways control a variety of cellular processes and have been linked to cell cycle regulation and survival (Levine and Kroemer, 2008). Previous reports have shown alterations in cell proliferation and apoptosis of distinct tumors after blocking CMA by LAMP-2A silencing (Kon et al., 2011; Saha, 2012; Zhou et al., 2016). Given LAMP-2A protein levels were reduced in melanoma cells expressing LAMP-2C, this led us to question whether cell proliferation or apoptosis was perturbed in these cells. DM331 2C myc cells exhibited alterations in the cell cycle distribution as monitored by flow cytometric analysis of cellular DNA content (**Figure 7A**). While the percentage of melanoma cells in G_0_/G_1_ phase decreased with ectopic LAMP-2C expression, an increase was detected in the percentage of these cells in G_2_/M phase (**Figure 7B**). A reduction in thymidine incorporation by DM331 2C myc cells was also detected compared to this cell transfected with vector alone (**Figure 7B**). Similarly, fewer DM331 cells expressing untagged LAMP-2C and SLM2-Mel 2C myc cells were at the G_0_/G_1_ stage, with these melanoma cells displaying more G_2_/M phase cells (**Figure 7C**). While differences in cell distributions in S phase were observed with altered LAMP-2C expression, these changes were variable among the different melanoma cells. These data suggest that LAMP-2C expression in these melanoma cells may disturb cell division via cell cycle arrest. To complement these studies, an analysis of melanoma cell death and necrosis was carried out using melanoma cells with and without ectopic LAMP-2C expression. Levels of apoptosis and/or necrosis were increased in each melanoma cell line with ectopic LAMP-2C expression compared to cells transfected with vector alone (**Figure 7D**). ROS generated by tumor cells can impact cellular autophagy pathways and growth (Poillet-Perez et al., 2015). ROS production was evaluated in melanoma cells with ectopic LAMP-2C and compared to the control melanoma cells with vector alone. There was no consistent increase or decrease in cellular ROS among the three pairs of tumor cell lines tested. Although a slight increase in ROS production was detected with DM331 2C myc cells compared to vector transfected cells, and a reduction in ROS production was observed for SLM2-Mel 2C myc cells compared to the vector transfected cells (**Figure 7E**). These results suggest that increased LAMP-2C expression in melanoma cells perturbs cell cycle progression as well as apoptosis and necrosis.

**FIGURE 8:**
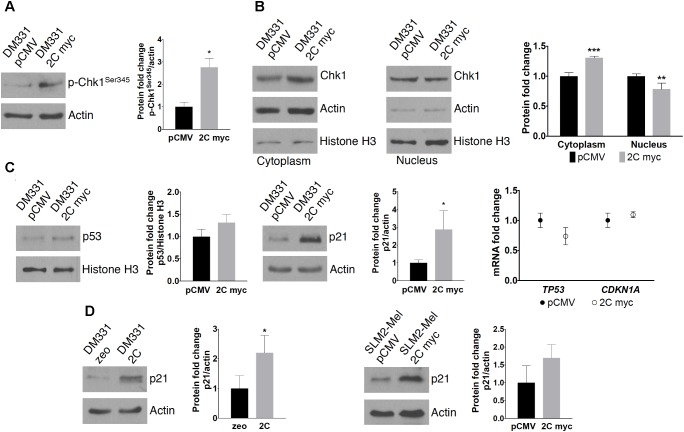
Effect of LAMP-2C on cell cycle regulators. **(A)** Chk1 activation was examined by detecting cellular levels of Chk1 phosphorylation at Ser345. **(B)** Chk1 subcellular localization was determined by extracting cytoplasmic and nuclear proteins from DM331 pCMV and DM331 2C myc cells. Protein levels were detected by western blotting. **(C)** Protein and mRNA levels of cell cycle regulators in DM331 pCMV and DM331 2C myc, p53 and p21, were examined by western blotting and qPCR. **(D)** Lysates from DM331 zeo and DM331 2C were resolved by SDS-PAGE and probed to detect p21 levels. Protein expression was quantified by densitometry and levels were normalized to actin levels. **(E)** Protein levels of p21 in SLM2-Mel pCMV and SLM2-Mel 2C myc were detected by western blotting. Protein expression was quantified by densitometry and levels were normalized to actin levels. Measurements in **(A–E)** represent relative values calculated by setting the results obtained for cells transfected with an empty vector equal to one for comparison to cells with ectopic LAMP-2C. Data were analyzed by two-way ANOVA or by two-tailed, unpaired Student’s *t*-test. ^∗^*p* < 0.05, ^∗∗^*p* < 0.01, and ^∗∗∗^*p* < 0.001 (*n* = 3).

Chaperone-mediated autophagy substrate Chk1, a key regulator during DNA replication and DNA damage responses, contributes to all cell cycle checkpoints, including G_1_/S, intra-S-phase, G_2_/M, and the mitotic spindle checkpoint (Patil et al., 2013). In response to genotoxic stress, Chk1 is phosphorylated and activates DNA damage responses to bring about cell cycle arrest, activate DNA repair pathways, and induce apoptosis when DNA damage is severe (Patil et al., 2013). Chk1 Ser345 phosphorylation is critical for this activation and function in response to DNA damage (Patil et al., 2013; Goto et al., 2015). Higher cellular levels of Chk1 Ser345 phosphorylation were detected in DM331 2C myc cells compared to control cells, suggesting increased activation of Chk1 in melanoma cells with high LAMP-2C expression (**Figure 8A**). Although Chk1 is mainly expressed in the nucleus, following activation Chk1 shuttles between the nucleus and cytoplasm (Patil et al., 2013; Goto et al., 2015). Consistent with the increased phosphorylation of Chk1 in cells with ectopic LAMP-2C, slightly more Chk1 protein was detected in the cytoplasm of these cells (**Figure 8B**). The tumor suppressor protein p53 and the cyclin-dependent kinase inhibitor p21 play important roles in G_1_ and G_2_ checkpoints (Giono and Manfredi, 2006; Karimian et al., 2016). Furthermore, increased cellular levels of p53 and p21 have been observed in cancer cells with LAMP-2A downregulation (Kon et al., 2011; Zhou et al., 2016). While p53 protein levels were slightly increased compared to control melanoma cells, cellular levels of p21 were markedly increased in DM331 cells with increased LAMP-2C expression (**Figure 8C**). Protein levels of p21 were also increased in DM331 cells expressing untagged LAMP-2C and SLM2-Mel 2C myc cells (**Figures 8D,E**). Changes in cellular levels of p53 and p21 were not a direct result of altered levels of p53 and p21 mRNA transcripts in DM331 cells with ectopic LAMP-2C myc expression (**Figure 8C**). Thus, enhanced LAMP-2C expression induces cell cycle arrest and affects survival by altering the abundance and activation of key cell cycle regulators.

### LAMP-2C Expression Reduces Melanoma Cells Tumorigenic Potential

**FIGURE 9:**
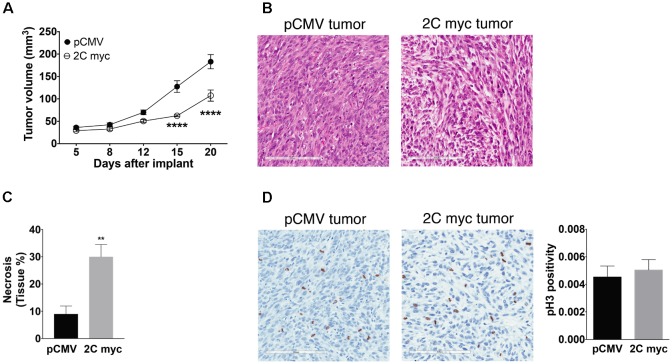
Ectopic expression of LAMP-2C decreased tumor growth in a xenograft mouse model. NSG mice were implanted subcutaneously in the flanks with 5 × 10^5^ DM331 cells with or without ectopic LAMP-2C myc expression. **(A)** Tumor growth was monitored biweekly (*n* = 13 per group). **(B)** Representative HE staining of primary control tumor or primary tumor with high ectopic expression of LAMP-2C myc. **(C)** Percentage of necrotic areas of primary tumors were evaluated by HE staining (*n* = 5 per group). **(D)** Representative pH3 staining of primary tumor with or without LAMP-2C myc expression. To determine changes in mitosis, pH3 positive cells were quantified in five hot spots areas of primary tumors. pH3 positivity was quantified dividing the number of pH3 positively stained cells by the total number of cells in the selected areas (*n* = 6 per group). Data were analyzed by two-way ANOVA or by two-tailed, unpaired Student’s *t*-test. Error bars indicate mean ± SEM. ^∗∗^*p* < 0.01 and ^∗∗∗∗^*p* < 0.0001.

LAMP-2A knockdown in cancerous cells has been documented to reduce tumorigenic capability and metastatic capacity (Kon et al., 2011; Zhou et al., 2016). Given that *in vitro* studies here showed changes in the cell cycle of melanoma cells, the tumorigenicity of melanoma cells with enhanced LAMP-2C expression was examined *in vivo*. Previous reports have demonstrated NOD.Cg-*Prkdc^scid^Il2rg^tm1Wjl^*/SzJ (NSG) mouse model provide an excellent *in vivo* system to assess human melanoma metastasis without the complication of host immune responses to tumor (Quintana et al., 2012). Here, NSG mice were injected in the flanks with DM331 cells with or without ectopic LAMP-2C myc, and animals monitored for tumor growth followed by sacrifice 20 days post tumor implant. Subcutaneous xenografts growth was reduced for tumors with high LAMP-2C myc (**Figure 9A**). Histology of primary tumors established differences in anatomy (**Figure 9B**). While melanoma cells from control tumor were spindle-shaped, the LAMP-2C myc tumor cells were epithelial-shaped and loosely joined together (**Figure 9B**). In addition, HE staining revealed necrotic areas in LAMP-2C myc tumors were three times greater than control tumors (**Figure 9C**). To examine tumor cell mitosis, tissue sections from palpable tumors where stained to detect phospho-Histone H3 (**Figure 9D**). While differences in cell density were again observed in comparing tumors with ectopic LAMP-2C or vector alone, no significant difference was detected in phospho-Histone H3 staining. Together these results revealed a novel role for LAMP-2C in diminishing melanoma growth *in vivo*.

## Discussion

Deregulation of autophagy pathways has been associated with melanoma development and progression. While immunochemistry of normal human melanocytes revealed low expression of LC3 protein, a histological MA marker, focal staining of LC3 molecules increased in spreading subcutaneous melanoma consistent with increased tumor MA (Checinska and Soengas, 2011; Corazzari et al., 2013). Immunohistochemical analysis of early and late stage melanomas revealed that late stage tumors associated with poor prognosis, expressed reduced levels of p62, a protein whose turnover is linked to enhanced MA (Ellis et al., 2014). High levels of LAMP-2A, a marker for CMA were detected in human melanoma biopsies compared with healthy skin, and reductions in LAMP-2A expression slowed murine melanoma growth *in vitro* (Kon et al., 2011). Such results suggest that pathways or proteins linked to autophagy may influence melanoma cell growth and tumor progression.

Here, studies examined the role of a lysosomal membrane protein, LAMP-2C in modulating autophagy as well as cell cycle and growth in several human melanoma cell lines. LAMP-2C is highly homologous to LAMP-2A and LAMP-2B, which regulate CMA and MA respectively (Eskelinen et al., 2005). While the three LAMP-2 isoforms are derived from a common precursor mRNA, differential expression of these isoforms has been observed. LAMP-2A expression levels and basal CMA activity were increased in a variety of human solid tumors, including melanoma, lung, breast, and gastric cancers (Kon et al., 2011; Saha, 2012; Zhou et al., 2016). Inhibition of the proteasome or MA, has been reported to increase LAMP-2A expression in neural cells (Yang et al., 2013). In contrast with *LAMP2A* and *LAMP2B* mRNA which are broadly expressed in different tissues, *LAMP2C* mRNA has a more limited tissue distribution (Perez et al., 2016). The mRNA for all three *LAMP2* isoforms increased in B lymphoblasts exposed to toll receptor ligands, which are associated with microbial infection (Perez et al., 2016). In the current study, treatment of melanoma cells with the pro-inflammatory cytokine IFN-γ significantly increased *LAMP2C* mRNA abundance with only marginal or no change in *LAMP2A* and *LAMP2B* mRNA. This may be due to an initial increase in the abundance of the *LAMP2* precursor mRNA with selective regulation of mRNA splicing or preferential mRNA stabilization to yield increased *LAMP2C* mRNA. The results with interferon-treated cells are also consistent with tissue or cell type specific differences in LAMP-2 isoform expression. The molecular mechanisms which control the expression of individual *LAMP2* mRNAs have not been well examined. As discussed below, post-transcriptional events can also regulate LAMP-2 protein expression and function.

Ectopic expression of LAMP-2C in melanomas disrupted CMA, as indicated by the accumulation of several proteins typically degraded by CMA including Chk1, IκBα, and p21 (Cuervo et al., 1998; Park et al., 2015; Zhang et al., 2018). Studies have described an intricate cross-communication and compensatory mechanisms among the different autophagic pathways and the proteasome (Park and Cuervo, 2013). Furthermore, several CMA protein substrates, including Chk1 and IκBα, can also be targeted for proteasome degradation in some cell types (Alkalay et al., 1995; Zhang et al., 2005). The current study examined whether cellular proteasome activity was decreased with increased LAMP-2C expression in tumors. Proteasome activity analysis revealed a slight increase in the activity of this enzyme in melanoma cells with increased LAMP-2C compared to control cells. Thus, it does not appear that increasing melanoma cell LAMP-2C expression, disrupts proteasome function. These findings are also consistent with previous reports demonstrating upregulation of proteasome activity in cancer cells with compromised CMA (Kon et al., 2011). Decreased LAMP-2A and LAMP-2B protein levels were observed in melanoma cells with ectopic LAMP-2C expression in the current study. Work by others has shown that reductions in cellular LAMP-2A levels blocks CMA and promotes accumulation of CMA substrates (Zhou et al., 2005, 2016; Kon et al., 2011). Levels of *LAMP2A* mRNA were unchanged in melanoma cells with ectopic LAMP-2C, suggesting alterations in post-transcriptional regulation of LAMP-2A molecules. Studies of several lysosomal and cytoplasmic proteases known to function in the turnover of LAMP-2A, failed to reveal a clear change in these enzymes that might account for the reduction in cellular LAMP-2A. Instead, attempts to stabilize LAMP-2A using protease inhibitors in cells with ectopic LAMP-2C, resulted in greater reductions in LAMP-2A abundance. While not previously linked to LAMP-2 stability, proteasome activity did increase in melanomas with ectopic LAMP-2C. LAMP-2A molecules also form oligomers in lysosomes which regulate CMA, and it is possible that increased LAMP-2C expression may perturb oligomer formation. Attempts to detect a physical association between LAMP-2A and LAMP-2C in melanoma cells, have not been successful to date. Post-translational modifications of LAMP-2 isoforms including glycosylation and phosphorylation have been reported (Tan et al., 2016; Li et al., 2017), and such modifications could be altered in cells with high levels of LAMP-2C. The SDS-PAGE mobility of LAMP-2A protein from cells with or without ectopic LAMP-2C was similar. Further studies will be necessary to examine the mechanisms influencing LAMP-2A protein abundance and structure in melanomas with increased LAMP-2C.

Increased expression of LAMP-2C in human melanomas also disrupted basal levels of MA as assessed by reduced autophagic flux and autophagosome abundance. Shifts in cancer cell metabolism coupled with changes in the tumor microenvironment can lead to increased hypoxia, nutrient and growth factor deprivation which induce MA (Morselli et al., 2009; Choi, 2012). Melanomas with increased LAMP-2C expression displayed reduced MA induction compared to control cells in response to serum starvation, a form of nutrient stress which typically upregulates MA in tumors to promote survival. As indicated, ectopic expression of LAMP-2C in melanomas reduced cellular levels of LAMP-2B protein with very modest decreases in *LAMP2B* mRNA. Little is known regarding the stability, post-translational modification, or turnover of LAMP-2B. Mutations in LAMP-2B were found in patients with Danon disease and associated with disruptions in MA flux (Crotzer et al., 2010). Results in the current study suggest that manipulating melanoma LAMP-2C expression may offer a novel means to disrupt basal and induced MA as well as CMA in melanomas.

A common feature in many human cancers is disruption of target genes involved in cell cycle progression and apoptosis. Lung and gastric cancer cells with compromised CMA activity exhibited increased levels of cell senescence regulators, such as p53 and p21 (Kon et al., 2011; Zhou et al., 2016). While reduced cell proliferation in lung cancer cells was not linked to cell cycle arrest, gastric cells with LAMP-2A knockdown displayed cell cycle arrest (Kon et al., 2011; Zhou et al., 2016). For murine LAMP-2A deficient fibroblasts cell cycling appeared unchanged, yet inducing DNA damage in these cells with etoposide or irradiation increased the percentage of cells in G_1_ and G_2_ while reducing cells in S phase (Park et al., 2015). In the current study, increased LAMP-2C levels in human melanomas cells induced cell cycle checkpoint and DNA damage responses as suggested by changes in cell cycle distribution (increased G_2_ and reduced G_1_ phase cells) with elevated cellular levels of p21 and activated phospho-Chk1 (Ser345). In melanoma cells with ectopic LAMP-2C, Chk1 protein abundance increased twofold while phospho-Chk1 levels were nearly threefold higher compared with cells transfected with vector alone. This may reflect the importance of CMA in the turnover of Chk1 in melanoma cells coupled with stress induced activation of Chk1. By contrast, induction of DNA damage in murine embryonic fibroblasts from Atg7- or Atg5-deficient animals with impaired MA, revealed an increase in proteasome activity, no change in total Chk1 protein levels, and a significant reduction in phospho-Chk1 (Ser345) (Liu et al., 2015). The cell cycle regulator p53 is well known to induce the expression of p21 (Giono and Manfredi, 2006), yet only a slight increase in p53 protein levels was seen in cells with LAMP-2C expression. p53 is targeted for degradation by the proteasome and CMA, dependent on p53 structure and mutations as well as levels of cellular CMA (Vakifahmetoglu-Norberg et al., 2013). Whether elevated protein levels of p21 are induced by a p53-dependent or -independent manner in these melanomas remains to be determined and is beyond the scope of the current study. The detection of increased phospho-Chk1 and p21 in melanoma cells with ectopic LAMP-2C was consistent with increased cell stress, potentially associated with activation of ROS production and/or DNA repair mechanisms. Measurements of ROS levels in melanomas with ectopic LAMP-2C did not reveal a consistent change compared to control cells. In response to DNA damage, Chk1 is phosphorylated at Ser345/Ser317. This activated phospho-Chk1 shifts its localization within the nucleus with some molecules moving into the cytoplasm (Wang et al., 2012). Consistent with this, experiments here revealed increased Chk1 in the cytoplasm of cells with ectopic LAMP-2C compared to the parental melanoma cells. Phospho-Chk1 in the nucleus as well as the cytoplasm appears to modulate distinct cell checkpoint events. Studies by Wang et al. (2012) demonstrated diminished cell viability for Chk1 mutant proteins with increased cytoplasmic residence.

Xenograft studies revealed LAMP-2C expression in melanoma cells reduced melanoma growth *in vivo*. Melanoma xenografts with high LAMP-2C cellular levels also displayed increased necrosis, changes in cell morphology, and less cell density in palpable tumors in stained tissue sections. The increased necrosis detected *in vivo* in tumors expressing LAMP-2C, was consistent with increased necrosis and apoptosis observed in melanoma cells with ectopic LAMP-2C *in vitro*. Immunohistochemistry was used to examine levels of mitosis *in vivo* for tumor cells with and without ectopic LAMP-2C. While no difference in phospho-Histone H3 was detected in this analysis, differences in tumor cell morphology and density were again apparent. An analysis of the effects of LAMP-2A knockdown on lung tumor cells did not reveal consistent increases in cellular apoptosis compared to LAMP-2A sufficient cells *in vitro*, however necrosis and reduced cell proliferation were noted for xenografts of human lung tumors with LAMP-2A knockdown (Kon et al., 2011). Together, the results in this report demonstrate ectopic expression of LAMP-2C in melanomas disrupted multiple cellular autophagy pathways, as well as cell cycle progression and survival. While the reductions in cell growth and increased p21 levels in these melanoma cells were consistent with decreased CMA and reduced expression of LAMP-2A, the melanoma cells with increased LAMP-2C did exhibit some unique differences. These include minimal changes in p53 protein levels, reduced LAMP-2B expression, reduced MA, cell cycle arrest, and high levels of Chk1 and phospho-Chk1. Increased apoptosis and necrosis were detected for melanomas with increased LAMP-2C expression *in vitro* and *in vivo*. In pilot studies, a highly aggressive triple negative breast tumor line TMD-231 was also transfected to increased LAMP-2C expression. No changes in the cell cycle or thymidine incorporation were observed with the breast tumor line with or without ectopic LAMP-2C. Thus, additional studies will be necessary to determine if LAMP-2C expression can modulate tumor growth and survival beyond melanoma lines. Given the complexity of cellular changes associated with LAMP-2C, it may be difficult to definitively pinpoint whether disruptions in autophagy pathways were linked to alterations in cell cycle and survival. These studies do, however, highlight a potential role for LAMP-2C as a tumor suppressor, which might be exploited to halt melanoma progression.

## Author Contributions

LP, GS, KP, and JB designed the experiments and interpreted the data. LP, AS, and GS performed the experiments. LP and JB wrote the manuscript. All the authors read and approved the manuscript.

## Conflict of Interest Statement

The authors declare that the research was conducted in the absence of any commercial or financial relationships that could be construed as a potential conflict of interest.

## Abbreviations

Chk1: checkpoint kinase 1
CMA: chaperone-mediated autophagy
CQ: chloroquine
HSC70: heat shock cognate protein 70
HSP90: heat shock protein 90
IFN-γ: interferon-gamma
LAMP: lysosome-associated membrane protein
LC3: microtubule-associated protein light chain 3
MA: macroautophagy
ROS: reactive oxygen species
